# Editorial: Antimicrobial resistance and therapy in critically ill patients

**DOI:** 10.3389/fmed.2023.1283865

**Published:** 2023-09-15

**Authors:** Qi Li, Shun-Jin Zhao, Jian-Cang Zhou

**Affiliations:** ^1^Department of Emergency Medicine, Lanxi People's Hospital, Lanxi, China; ^2^Department of Respiratory and Critical Care Medicine, Lanxi People's Hospital, Lanxi, China; ^3^Department of Critical Care Medicine, Sir Run Run Shaw Hospital, Zhejiang University School of Medicine, Hangzhou, China

**Keywords:** antimicrobial resistance, critically ill, therapy, carbapenem-resistant organisms, carbapenem-resistant *Klebsiella pneumoniae*

Multidrug-resistant (MDR) microbes infection for critically ill patients is a big challenge in clinical practice and is associated with greatly increased mortality (1). This Research Topic includes three articles that explored the clinical and microbiological characteristics of MDR pathogen infection in patients admitted to intensive care units (ICU) in China (Wu H. et al., Wu H.-N. et al., Li et al.).

Wu H.-N. et al. retrospectively analyzed the distribution and antibiotic resistance of pathogens based on the clinical data of intensive care patients with bloodstream infections presented to a Chinese tertiary hospital and explored the value of procalcitonin (PCT) for the differentiated diagnosis of bloodstream infections caused by various pathogens. Gram-negative bacteria were the most frequently isolated microorganisms and were associated with a higher percentage of complications such as brain dysfunction, acute kidney injury, and thrombocytopenia. It was observed that PCT was a not good biomarker to distinguish bloodstream infections caused by various pathogens or fungi. Given that the mortality for patients with carbapenem-resistant *Klebsiella pneumoniae* (CRKP) bloodstream infection is reported to be as high as 30%−70%, Wu H. et al. used a logistic analysis to assess the association between the neutrophil-to-lymphocyte ratio (NLR) on 4th-day and 28th-day mortality. After balancing the confounders, NLR on the 4th day was associated with the 28th-day mortality, whereas the appropriate initial therapy was an independent protective factor. Moreover, the authors suggested that the trend of the NLR during therapy may help to evaluate the efficacy of different anti-infection therapy strategies at an early stage. In another article, Li et al. described their experience in the management of post-neurosurgical central nervous system infection caused by MDR Gram-negative bacteria with combined intraventricular and intravenous polymyxin B administration. After a mean duration of 14 days of treatment, all six cases caused by CRKP or carbapenem-resistant *Acinetobacter baumannii* (CRAB) were cured and no obvious kidney injury occurred.

This Research Topic also includes three articles on the superinfection of patients with SARS-CoV-2 infection who were admitted to the ICU (Karlsson et al., Yoon et al., Casarotta et al.). Yoon et al. demonstrated that bacterial superinfections were common in a tertiary Korean academic hospital and that more than one-third of the bacterial superinfection cases were caused by multidrug-resistant pathogens. Moreover, bacterial superinfection was associated with significantly fewer ventilator-free days, longer ICU and hospital stays. As many studies reported that a CRAB-associated bloodstream infection was the crucial risk factor for death in patients with COVID-19 (2), Casarotta et al. compared two different antibiotic strategies for CRAB infection in terms of microbiological negativization. The *Protocol* group, which was managed with combination therapy of nebulized and intravenous colistin, high-dose tigecycline, and high-dose ampicillin/sulbactam, was associated with a significantly higher microbiological clearance compared to the *Control* group, which consisted of patients treated with a combination of two antibiotics (100% vs. 36.4% respectively). In a prospective longitudinal study in Sweden conducted by Karlsson et al., the authors evaluated the complicated bacteriuria and antibiotic resistance for ICU-admitted COVID-19 patients. They found that the vast majority of patients received antibiotics on ICU admission. Longer stays in ICUs linearly correlated with bacteriuria, and the authors proposed that biofilms in urinary catheters act as a reservoir of pathogenic bacteria with the potential to develop and disseminate antibiotic resistance.

This Research Topic also comprises an uncommon but interesting study by Bushuven et al. to evaluate the feasibility of hand hygiene in a manikin cardiopulmonary resuscitation (CPR) study, given that CPR scenarios are at high risk for healthcare-associated infections. By studying Advanced Cardiovascular Life Support (ACLS) courses in a manikin simulation, they found more than half of hand-cleaning indications could have been accomplished without delaying patient resuscitation and they concluded that hand disinfection can be implemented without compromising quality in acute care. In patients with severe acute pancreatitis (SAP), secondary MDR pathogen infection plays a vital role in increased mortality and prolonged hospital and ICU stays (3). MDR pathogen infection in patients with SAP is lethal and generally associated with excessive antibiotic exposure. As shown in the study by Shajiei et al., which used a previously reported SAP trial data, PCT-guided antibiotics management significantly reduced antibiotic usage but it did not translate into a detectable change in antimicrobial resistance.

This Research Topic also includes an original study by Byrnes et al. that aimed to identify the optimal tissue source of both naïve and cytokine pre-activated mesenchymal stromal cells (MSCs) to enhance the resolution of late-phase organizing pneumonia caused by CRKP. Organizing pneumonia is a pattern of lung-tissue repair after injury and it can be cryptogenic or a response to a specific lung injury in many diverse clinical contexts. Given the therapy for organizing pneumonia is empirical and few therapies have been confirmed besides systemic glucocorticoid therapy (4), they demonstrated that delayed MSC therapy enhanced the resolution of lung injury induced by CRKP infection and favorably modulated immune cell profile, which indicates the potential role of MSC to facilitate the resolution of pulmonary organization after MDR pathogen infection.

## Author contributions

QL: Writing—original draft. S-JZ: Writing—review and editing. J-CZ: Writing—original draft, Writing—review and editing.
